# The Experience and the Characteristics of Patients With Tele-ICU Implementation in a Rural Community Hospital

**DOI:** 10.7759/cureus.41971

**Published:** 2023-07-16

**Authors:** Yoshiaki Iwashita, Ayana Ishigame-Kitayama, Akitaka Yamamoto, Kyohei Itoh, Masako Takenaka, Shinnnosuke Morimoto, Yasuhisa Yamamoto

**Affiliations:** 1 Department of Emergency and Critical Care Medicine, Shimane University, Izumo, JPN; 2 Department of Rehabilitation Medicine, Wakayama Medical University, Wakayama, JPN; 3 Department of Orthopedics, Naga Municipal Hospital, Kinokawa, JPN; 4 Department of Pediatrics, Wakayama Medical University, Wakayama, JPN; 5 Department of Internal Medicine, NachiKatsuura Town Onsen Hospital, NachiKatsuura, JPN; 6 Department of Internal Medicine, Onodani Clinic, Kiho-cho, JPN

**Keywords:** intensive care unit, internal medicine, telemedicine, tele-icu, community emergency department, internal medicine in rural areas, general critical care, telemedicine (tm)

## Abstract

Background

Tele-ICUs are increasingly being used in the US. In Japan, young physicians mainly operate rural community hospitals to manage severely ill patients. However, the introduction of the tele-ICU system in Japan is still uncommon. We introduced a tele-ICU system to a community hospital. The objective of this study is to determine if the newly introduced tele-ICU system is being effectively utilized.

Methods

This is a single-center, retrospective observational study. We introduced the tele-ICU system to the NachiKatsuura Town Onsen Hospital, Japan, in 2019. Thereafter, we retrospectively explored the characteristics of the consulted cases, the time of consultation, the Sequential Organ Failure Assessment (SOFA) score, and the number of consultations by month from 1st July 2019 to 31st March 2020. The main outcome was the monthly number of consultations, and other measures included the clinical characteristics of the consulted cases.

Results

A total of 81 cases were referred to the tele-ICU system within nine months. Sixty-two cases, excluding those with missing data, were included in the analysis. The number of consultations was almost constant during the study period and was most frequent during the day. The recommendations from tele-ICU physicians were mostly “advice on the treatment plan.” The mean SOFA score was 2.56.

Conclusions

We introduced a Japanese-type tele-ICU system for Japanese rural community hospitals. Many cases from rural community hospitals that were referred to the tele-ICU systems were moderately severe and did not require urgent transportation. These cases are not indicated for emergency transportation and should be treated in rural community hospitals.

## Introduction

With the increasing number of mechanically ventilated patients, the number of patients requiring intensive care is increasing [1]. Owing to the limited number of intensive care unit (ICU) beds, all potential ICU candidates cannot be admitted to ICUs worldwide [2]. Tele-ICU, which is a remote critical care system that connects off-site intensivists with ICU patients and on-site healthcare providers, is started to develop in recent years. In the US, tele-ICUs are implemented for critical care beds in rural hospitals and have decreased mortality, length of ICU stay, and ventilator days [3]. In Japan, the rate of ICU beds versus general beds is very low [4]. Therefore, many critically ill patients are treated in general wards and rural community hospitals [5,6]. In addition, the shortage of critical care physicians and emergency physicians poses a problem in small rural hospitals in Japan [7]. In Japan, the tele-ICU system is still uncommon; however, a small number of systems have been initiated [8]. Several Japanese community hospitals had introduced telemedicine. However, these systems were limited to specialties such as stroke and obstetrics/gynecology and were designed to allow tertiary hospitals to pick up patients with indications for transfer to higher hospitals. The real problem for physicians in community hospitals was how to manage patients in community hospitals who were not eligible for transfer to a higher-level medical institution. Therefore, the previous system was discontinued shortly after its introduction. Tele-ICU system is mainly focused on aiding physicians in rural hospitals. However, the Japanese tele-ICU system can be applied to critically ill admitted patients and also emergency room patients, because, under the Japanese medical specialist system, an intensivist is qualified after obtaining an emergency medicine specialist or anesthesiology specialist training. Therefore, most intensivists are board certified in emergency medicine. Thus, tele-ICU physicians in Japan can provide advice on consultations from the emergency room. We believed this Japanese-style tele-ICU system would be helpful and well utilized in rural community hospitals in Japan. Thus, we introduced Japanese intensivists-operated tele-ICU systems into rural community hospitals. The purpose of this study is to determine if the newly introduced tele-ICU system is being effectively utilized in rural community hospitals.

## Materials and methods

We introduced a tele-ICU system operated by T-ICU (Hyogo, Japan) in a rural community hospital in NachiKatsuura in July 2019. T-ICU is the name of a company that provides Japanese intensivist-provided tele-ICU systems. We retrospectively investigated the consulted cases in the tele-ICU between July 1, 2019, and March 31, 2020.

NachiKatsuura is a small city with a population of 15,000. NachiKatsuura Town Onsen Hospital has 120 general beds and no ICU beds. There is no board of critical care or emergency medicine physicians and no specialist nurses in critical care or emergency medicine. Currently, there is no certification system for respiratory therapists in Japan. The hospital is located approximately 150 km from the nearest tertiary emergency medical facility. In Japan, emergency helicopter transportation systems do not operate at night. Additionally, many physicians at the hospital are young as they have graduated from the newly established Regional Quota Admission System. Under this program, medical students are required to work at a local hospital for several years after their graduation.

One emergency and critical care physician (author YI) started working in a community hospital in 2017. He started to help the night shift at NachiKatsuura Town Onsen Hospital once a week. He noticed the difficulty and anxiety of young physicians, and he advised the young physicians. He also talked to the director of the hospital about introducing the T-ICU system for continuous advice for young physicians. The funding for the introduction was from hospital revenue.

A commercial company “T-ICU” provides tele-ICU services. The board of critical care physicians (this refers to the intensivist certified by the Japanese Society of Intensive Care Medicine) may be consulted at any time. Most critical care physicians are also affiliated with the Japanese Association for Acute Medicine-certified boards of emergency medicine. The consultation starts with phone calls from the local hospital physicians, and if the consultant or consultee decides to discuss in depth with electronic chart information, the consultee can mirror the electronic chart, including laboratory data, imaging, and chart information. The consultant can only advise local staff but cannot prescribe due to Japanese law. It is also not possible for tele-ICU physicians to view charts without approval from local hospitals.

The study included patients admitted to NachiKatsuura Town Onsen Hospital between July 2019 and March 2020, for whom patient consultations were conducted using T-ICU's tele-intensive care system. Exclusions included patients who do not have enough data on the electronic chart. We collected the consulted cases from the electronic charts and records in the T-ICU. Patients' age, sex, diagnostic category (system), and Sequential Organ Failure Assessment (SOFA) scores were collected from electronic charts. The time of consultation, recommendations from tele-ICU physicians, and information on how local hospital physicians acted were collected from the T-ICU consulted records.

The primary outcome of the evaluation of implementation effectiveness was defined as the number of consultations per month, and the secondary outcome was how the physicians in rural hospitals react to the recommendations from T-ICU.

This study was approved by the Shimane University Medical Research Ethics Committee (study number 4993; dated: November 6, 2020). The research title is "Epidemiological data of tele-ICU use in rural community hospitals." Informed consent was waived by showing the opt-out form. This study was followed in accordance with the Helsinki Declaration of 1975.

## Results

**Figure 1:**
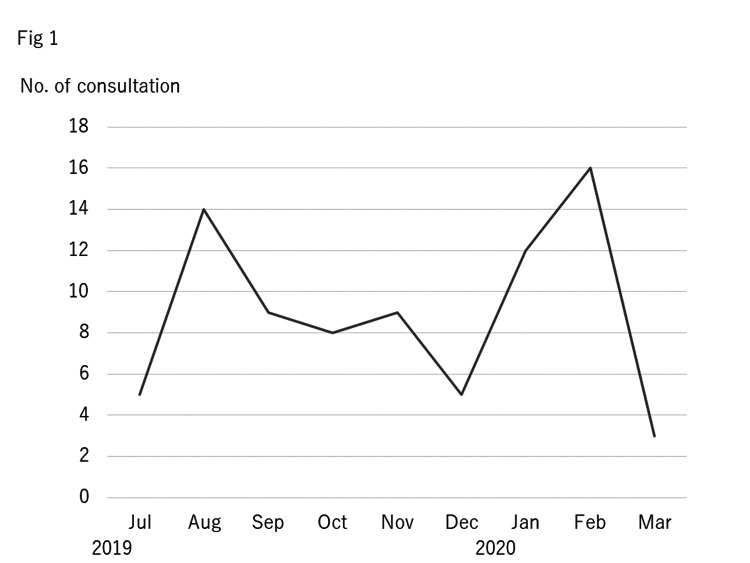
Number of consultations by months The monthly consultation numbers from the hospital are shown. X-axis shows the months, and Y-axis shows the number of consultations.

A total of 81 consultations were conducted between 1st July 2019 and 31st March 2020. Figure 1 shows the number of monthly consultations. The number of consultations was maximum in August and February and then minimum in March.

Of the 81 cases, some cases had several missing data, thus, we excluded 19 cases (laboratory data required for SOFA score calculations have not been obtained). Sixty-two cases were candidates for the following analysis. Table 1 presents the characteristics of the consultations. The mean patient age was 74.0 years. The time category of the consultation was mainly daytime (39, 62.9%) and lowest at late night (2, 3.2%).

**Table 1:** Characteristics of consultations

Characteristics	N = 62
Age (mean ± SD)	74.0 ± 18.3
Sex (male:female)	33:29
Consulted time category	
Day (8:01-17:00)	39 (62.9%)
Night (17:01-0:00)	21 (33.9%)
Late night (0:01-8:00)	2 (3.2%)
Recommendation	
Advice on the treatment plan	52
Recommend transfer	10

Tele-ICU physicians mostly recommended treatment plans in the hospital (52, 83.9%). Only 10 (16.1%) cases received transfer recommendations to higher hospitals. The number of system-wise consultation categories was the highest for respiratory and cardiac categories (Table 2). The mean SOFA score was 2.56 (Table 3).

**Table 2:** Consultation categories

Category	N = 62
Cardiac	13
Respiratory	16
Digestive	11
Neuro	7
Endocrine	2
Hematologic	1
Renal	0
Orthopediatrics	1
Ophthalmologic	0
Otorhinolaryngology	2
Dental and oral surgery	0
Dermatologic	2
Genitourinary	3
Obstetrics and gynecology	0
Other	4

**Table 3:** Severity of the patients Average of SOFA scores for each organ and the average of SOFA scores for the total. SOFA: Sequential Organ Failure Assessment.

Organs	Mean SOFA score
Respiratory	1
Coagulation	0
Hepatic	0
Circulation	0
CNS	1
Renal	1
Total	2.56

Examples of the case

Cases consulted to the T-ICU included (1) those who were considered not suitable for transfer due to advanced age, and (2) those who were difficult to diagnose or to decide the treatment plan. Examples of the consulted cases are below.

Case 1

A 93-year-old female, admitted to a nursing home, who performed activities of daily living (ADL) on a wheelchair, was hospitalized for aspiration pneumonia. She weighed 25 kg, with SpO2 of 95% (O2 3 L/min), and had aortic valve regurgitation and mitral valve regurgitation. She was consulted for advice on how to administer intravenous fluids while fasting.

Suggestion: T-ICU suggested that the infusion volume be started at 1000-1500 ml/day and adjusted according to the urine volume.

Case 2

A 93-year-old woman was admitted to the hospital with septic shock due to a urinary tract infection. On admission, she had tachycardic atrial fibrillation and required noradrenaline 0.05 μg/kg/min. On day three of hospitalization, she was consulted because her blood test inflammatory response did not decrease.

Suggestions: T-ICU advised CT follow-up, change of antibiotics, and adjustment of infusion volume.

Case 3

A 60-year-old male was admitted four days earlier for a pit viper bite. No antiserum was administered because the grade was 2 at the time of admission. During the course of the hospitalization, the swelling worsened, so we consulted with the patient.

Suggestions: After confirming that there was no evidence of compartment syndrome or myonecrosis, T-ICU advised that the patient could be followed up without any additional treatment.

## Discussion

We explored the nine-month experience of implementing the tele-ICU system in rural community hospitals in Japan. There was constant consultation throughout the post-introduction period. This indicates that the community hospital's medical staff recognized the need for a tele-ICU system. The use of this system in Japan is not required by regulation and the costs of this system are incurred by hospitals without increasing medical fees, so community hospital doctors will not use it unless they feel it is useful. This hospital actually had a telemedicine system that was introduced in the past and was discontinued due to a lack of success in use. Therefore, the fact that this system is used consistently is considered to be an outcome of the satisfaction of the medical staff.

The tele-ICU system is a well-known system in the US [9-11], but it is still uncommon in Japan. The consultant physicians in the T-ICU were ICU board-certified physicians and were affiliated with the emergency medicine board of physicians. In Japan, intensive care physicians have a basic specialty on the board of emergency medicine or anesthesia; therefore, this is the most common form of intensivist in Japan. NachiKatsuura Town Onsen Hospital is a typical rural community hospital with a small number of young physicians managing hospitalized patients. Thus, although this was a single-center analysis, the current characteristics will be similar to that implemented in other community hospitals.

The mean SOFA score was 2.56, a majority of consultations were done in the daytime, and most recommendations by tele-ICU physicians dealt with advice on hospital management. These characteristics indicate that the consulted patients are sub-critically ill and do not require urgent transfer to higher hospitals. Local hospital physicians can directly consult and transfer critically ill patients to the nearest tertiary care institution. The main problem for local hospital physicians is the management of patients who are not indicated for transfer. The advice from the ICU and emergency medicine board physicians helps manage the needs of the local community hospital physicians.

Our report is unique because tele-ICU and emergency medicine consultations are implemented in community hospitals where there are no ICUs. In Japan, the number of board-certified emergency physicians and critical care physicians is low. Therefore, the emergency rooms or ICUs especially in rural hospitals are operated by other physicians such as general physicians, internal medicine physicians, and surgeons. T-ICU is a company where Japanese physicians operate tele critical care services. Since Japanese intensivists also hold emergency board certification, it was suitable for helping emergency rooms and critical care in rural hospitals. Reports from telemedicine use in emergency departments are increasing; one report indicates that most consultations facilitate rapid transfer [12], and another reported use is the screening of crowded emergency departments [13]. These studies are focused on overcrowded emergency departments, where the situation is different from that of local community hospitals. There are some reports researching the effect of the implementation of tele-ICU systems in small local hospitals [14,15]; however, these hospitals have ICUs and the ICUs and local community hospital systems differ between countries. Hence, our results apply to countries where the situation is similar to that of Japanese hospitals.

Our study has several limitations. First, this report does not include patient-centered outcomes, such as mortality, morbidity, and the number of transfers. The main target was moderately severe patients who are not indicated for transfer, because of old age. For those patients, the clinical outcomes should not only be survival, and if the patient and the family feel happy, the outcome might be better for those. Similarly, if the patient's clinical outcome did not change, young physicians may feel comfortable with specialized physicians. In addition, one of the objectives of implementing this system is not to reduce the number of transfers but rather to make them more appropriate. Appropriate consultation may either increase or decrease the number of transfers. Therefore, the primary outcome of this study is that local hospital physicians trust the tele-ICU system and the number of consultations is appropriate as an indicator of this. Second, it was difficult to calculate the percentage of patients consulted. The likely candidates for our study were all hospitalized and emergency department patients and the data were not available. However, the mean number of consultations was more than once a week, indicating that local physicians had sufficient access to evaluate the system. Third, the survey was conducted at a single center. The needs of rural hospitals may differ from those of districts. However, a shortage of physicians is a common problem in rural medicine in Japan. Young physicians work with a relatively low number of senior physicians. Thus, our results should be similar for many rural Japanese hospitals. Fourth, in Japan, medical treatment other than in person is prohibited by law in principle. As a result, providers of telemedicine cannot be proactively involved in medical treatment and do not have prescriptive authority. They can only consult with local medical professionals when they feel it is necessary. The service featured in this study, however, allows not only phone calls, but also video chat, electronic medical record screen sharing, and the ability to take a picture of the patient using video if the consultant shares the video, making it similar to telemedicine services used worldwide.

## Conclusions

We have reported the nine months history of tele-ICU system use in a rural community hospital. The consultations constantly appeared during the study period. The characteristics of the consulted cases were sub-critically ill.
